# The Effect of Complications on Oncological Outcomes of Colorectal Cancer Patients After Primary Surgery: A Propensity Score Matching Analysis

**DOI:** 10.3389/fonc.2022.857062

**Published:** 2022-06-03

**Authors:** Xiao-Yu Liu, Bin Zhang, Bing Kang, Yu-Xi Cheng, Chao Yuan, Wei Tao, Zheng-Qiang Wei, Dong Peng

**Affiliations:** ^1^ Department of Gastrointestinal Surgery, The First Affiliated Hospital of Chongqing Medical University, Chongqing, China; ^2^ Department of Clinical Nutrition, The First Affiliated Hospital of Chongqing Medical University, Chongqing, China

**Keywords:** colorectal cancer, outcome, complications, propensity score matching, surgery

## Abstract

**Purpose:**

The purpose of this study is to explore the oncologic outcomes of complications on colorectal cancer (CRC) patients who underwent primary surgery using a propensity score matching (PSM) analysis.

**Methods:**

A retrospective study was conducted from Jan 2011 to Jan 2020 in a clinical center. The overall survival (OS) and disease-free survival (DFS) were compared among the no complications group, the major complications group and the minor complications group.

**Results:**

A total of 4250 CRC patients who underwent radical primary surgery were included in the current study. Among them, 927 (21.8%) patients suffered complications. After 1:1 ratio PSM, there were 98 patients in the major complications group and in the minor complications group, and 911 patients in the overall complications group and in the no complications group. There was no significant difference in terms of baseline information after PSM (p>0.05). Complications were independent predictors of OS (p=0.000, HR=1.693, 95% CI=1.476-1.941) and DFS (p=0.000, HR=1.555, 95% CI=1.367-1.768). In terms of specific tumor stage, the no complications group had better OS on all stages (p=0.006) and stage III (p=0.003) CRC than the complications group after PSM. Furthermore, the no complications group had better DFS on all stages (p=0.005) and stage III (p=0.021) CRC than the complications group after PSM. However, there was no significant difference between the minor complications group and the major complications group in different tumor stages (p>0.05).

**Conclusion:**

Complications were associated with poor prognosis of CRC and surgeons should be cautious of the adverse events.

## Introduction

Colorectal cancer (CRC) is one of the most common cancers in the world (1). There were an estimated 1.09 million new cases in 2018 which accounted for 10% of all cancer cases (2). Although there were many treatments for CRC, radical resection was still the cornerstone (3–5).

Despite the experienced surgical technique, the implementation of modern perioperative programs and the improvement of surgical instruments, the incidence of complications is still high (6–8). Complications can increase hospital stay and costs (9). As was reported in previous studies, complications could affect the prognosis of digest cancers including esophageal cancer and gastric cancer (10, 11).

However, the effect of complications on CRC patients remained controversial. Most studies reported that complications were associated with poor prognosis (12–14), but few studies reported that complications had no impact on prognosis (15, 16). Therefore, the purpose of this study is to explore the oncologic outcomes of complications on CRC patients who underwent primary surgery.

## Methods

A retrospective study was conducted from Jan 2011 to Jan 2020 in a single clinical center. This study was in accordance with the World Medical Association Declaration of Helsinki. Ethical approval was obtained from the Institutional Ethics Committee of the First Affiliated Hospital of Chongqing Medical University (2021-537), and all patients signed informed consents.

### Patient`s Selection

**Figure 1:**
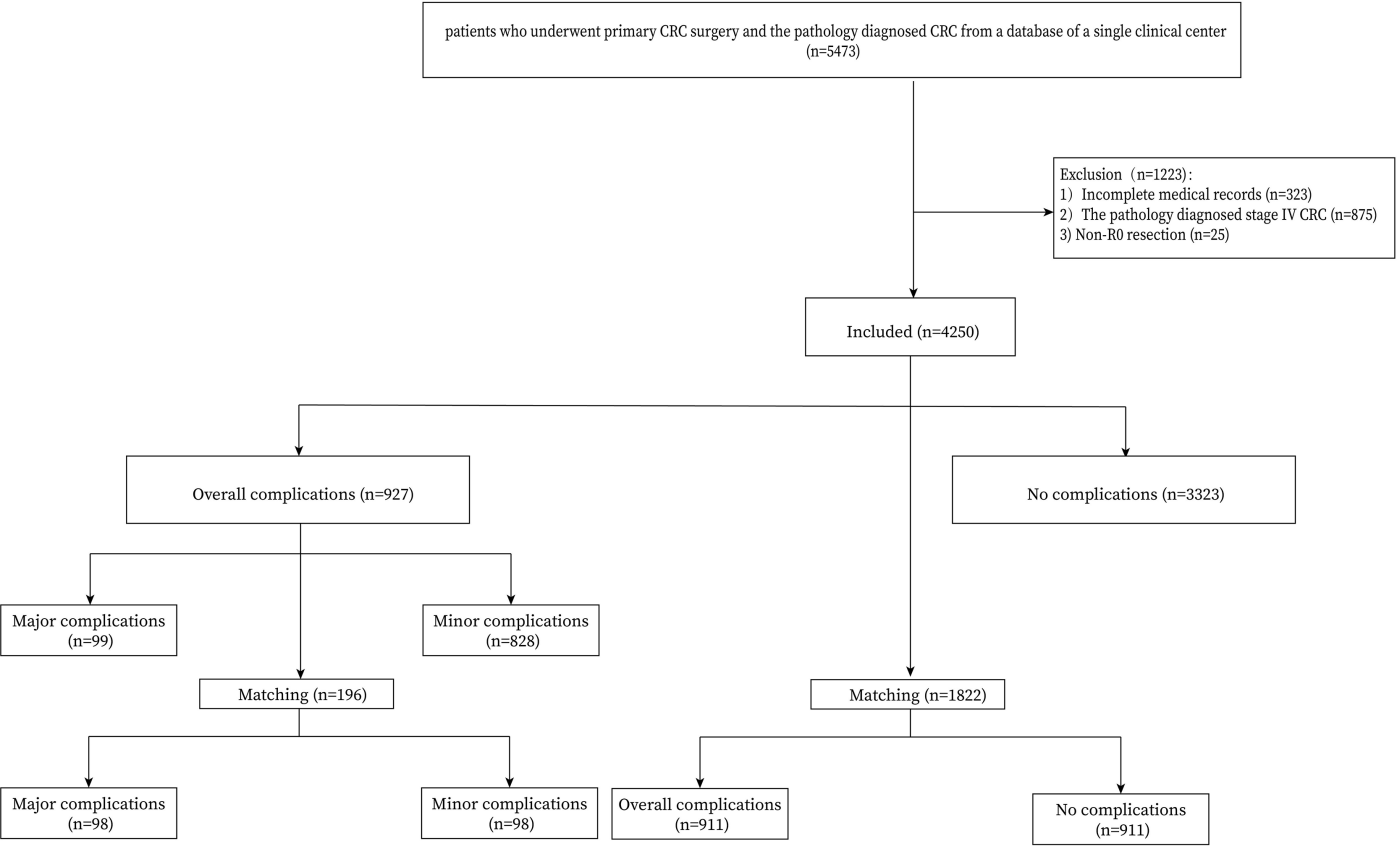
Flow chart of patient selection.

Patients who underwent primary CRC surgery and confirmed by pathology were included in this study (n=5473). The exclusion criteria were as follows: 1, incomplete perioperative medical information (n=323); 2, stage IV CRC (n=875); and 3, Non-R0 CRC surgery (n=25). Finally, 4250 CRC patients were identified from the database of a single clinical center and the flow chart was shown in **Figure 1**.

### Surgery Management

We performed radical CRC surgery which was according to the principles of oncology. Total mesorectal excision or complete mesocolic excision was performed, and the pathology confirmed R0 resection. The American Joint Committee on Cancer suggested a minimum of 12 lymph nodes to be assessed in the surgical specimen to verify lymph-node negative disease (17). Patients were regularly followed-up every three months for the first three years and six months for the following two years.

### Definition

The tumor stage was diagnosed according to the AJCC 8^th^ Edition (18). Complications were defined according to the Clavien-Dindo classification (19), major complications were defined as ≥ III classification complications and minor complications were defined as I or II classification complications. Overall survival (OS) was defined as the time from radical CRC surgery to the time of death, last follow-up or lost follow-up. Disease-free survival (DFS) was defined as the time from radical CRC surgery to the time of recurrence, death, last follow-up or lost follow-up.

### Data Collection

We collected the medical information from the inpatient system, outpatient system and through telephone reviews. The baseline information which included age, sex, body mass index (BMI), patients’ habit (smoking and drinking), comorbidity (coronary heart disease (CHD), type 2 diabetes mellitus (T2DM) and hypertension), surgery history, neoadjuvant therapy, surgical methods, protective stoma, tumor location and tumor stage were collected.

### Propensity Score Matching (PSM)

PSM was conducted between the complications group and the no complications group, and between the major complications group and the minor complications group. Nearest neighbor matching was performed without replacement at a 1:1 ratio and a caliper width with a 0.01 standard deviation was specified. The following baseline information were matched:age, sex, BMI, smoking, drinking, surgery history, hypertension, T2DM, CHD, neoadjuvant therapy, surgical methods, protective stoma, tumor location and tumor stage.

### Statistical Analysis

Continuous variables are expressed as the mean ± SD and independent-sample t test was used to analyze the difference between the complications group and the no complications group, and between the major complications group and the minor complications group. Frequency variables are expressed as n (%), and Chi-square test was used. The Kaplan-Meier curve was conducted to compare no complications/overall complications/major complications/minor complications on different tumor stages, and cox regression analyses were performed to identify independent predictive factors for OS and DFS. Data were analyzed using SPSS (version 22.0) statistical software. A bilateral p value of <0.05 was considered statistically significant.

## Results

### Patients

A total of 4250 CRC patients who underwent radical primary surgery were included in the current study. Among them, 927 (21.8%) patients suffered complications. PSM was conducted between the complications group and the no complications group, and between the major complications group and the minor complications group. After 1:1 ratio PSM, there were 98 patients in the major complications group and in the minor complications group, and 911 patients in the overall complications group and in the no complications group (**Figure 1**).

### Baseline Characteristics of Included Patients Before and After PSM

Baseline information were compared between the complications group and the no complications group. The complications group had older age (p=0.000), higher rate of smoking (p=0.042), surgery history (p=0.002), hypertension (p=0.000), T2DM (p=0.000) and CHD (p=0.000), and lower rate of laparoscopic CRC surgery (p=0.000) and neoadjuvant therapy (p=0.006) before PSM. After 1:1 ratio PSM, there were no significant difference between the complications group and the no complications group (p>0.05) in terms of baseline information (**Table 1**).

**Table 1:** Baseline characteristics of included patients before and after PSM.

Characteristics	Before PSM			After PSM		
	Complications (927)	No complications (3323)	P value	Complications (911)	No complications (911)	P value
Age (year)	65.6 ± 12.5	62.2 ± 11.9	0.000*	65.3 ± 12.5	65.3 ± 11.4	0.925
Sex			0.140			0.630
Male	564 (60.8%)	1932 (58.1%)		554 (60.8%)	564 (61.9%)	
Female	363 (39.2%)	1391 (41.9%)		357 (39.2%)	347 (38.1%)	
BMI (kg/m^2^)	22.5 ± 3.4	22.7 ± 3.2	0.052	22.5 ± 3.3	22.4 ± 3.2	0.433
Smoking	377 (40.7%)	1230 (37.0%)	0.042*	369 (40.5%)	375 (41.2%)	0.775
Drinking	289 (31.2%)	638 (19.2%)	0.673	283 (31.1%)	283 (31.1%)	1.000
Surgery history	252 (27.2%)	743 (22.4%)	0.002*	240 (26.3%)	236 (25.9%)	0.831
Hypertension	290 (31.3%)	818 (24.6%)	0.000*	278 (30.5%)	260 (28.5%)	0.355
T2DM	149 (16.1%)	372 (11.2%)	0.000*	138 (15.1%)	152 (16.7%)	0.370
CHD	59 (6.4%)	120 (3.6%)	0.000*	55 (6.0%)	49 (5.4%)	0.545
Neoadjuvant therapy	34 (3.7%)	199 (6.0%)	0.006*	34 (3.7%)	36 (4.0%)	0.807
Protective stoma	39 (4.2%)	180 (5.4%)	0.141	39 (4.3%)	32 (3.5%)	0.397
Laparoscopy	700 (75.5%)	2908 (87.5%)	0.000*	699 (76.7%)	701 (76.9%)	0.912
Tumor location			0.373			0.707
Colon	416 (44.9%)	1546 (46.5%)		409 (44.9%)	417 (45.8%)	
Rectum	511 (55.1%)	1777 (53.5%)		502 (55.1%)	494 (54.2%)	
Tumor stage			0.303			0.398
I	193 (20.8%)	657 (19.8%)		190 (20.9%)	176 (19.3%)	
II	379 (40.9%)	1453 (43.7%)		369 (40.5%)	397 (43.6%)	
III	355 (38.3%)	1213 (36.5%)		352 (38.6%)	338 (37.1%)	

Variables are expressed as the mean ± SD, n (%), *P-value <0.05.

T2DM, type 2 diabetes mellitus; BMI, body mass index; PSM, propensity score matching; CHD, coronary heart disease.

### Baseline Characteristics of Complications Before and After PSM

We compared baseline information between the major complications group and the minor complications group. The major complications group had higher portion of males (p=0.005), drinking (p=0.020) and rectal cancer (p=0.001) before PSM. After 1:1 ratio PSM, no significant difference was found in the two groups (p>0.05) (**Table 2**).

**Table 2:** Baseline characteristics of complications before and after PSM.

Characteristics	Before PSM			After PSM		
	Major complications (99)	Minor complications (828)	P value	Major complications (98)	Minor complications (98)	P value
Age (year)	67.0 ± 11.4	65.4 ± 12.6	0.243	66.8 ± 11.4	66.3 ± 12.3	0.754
Sex			0.005*			0.621
Male	73 (73.7%)	491 (59.3%)		72 (73.5%)	75 (76.5%)	
Female	26 (26.3%)	337 (40.7%)		26 (26.5%)	23 (23.5%)	
BMI (kg/m^2^)	22.8 ± 3.5	22.5 ± 3.3	0.361	22.8 ± 3.5	23.6 ± 3.3	0.105
Smoking	47 (47.5%)	330 (39.9%)	0.145	47 (48.0%)	41 (41.8%)	0.389
Drinking	41 (41.4%)	248 (30.0%)	0.020*	40 (40.8%)	36 (36.7%)	0.558
Surgery history	23 (23.2%)	229 (27.7%)	0.350	23 (23.5%)	24 (24.5%)	0.867
Hypertension	31 (31.3%)	259 (31.3%)	0.995	31 (31.6%)	33 (33.7%)	0.761
T2DM	17 (17.2%)	132 (15.9%)	0.753	17 (17.3%)	23 (23.5%)	0.288
CHD	7 (7.1%)	52 (6.3%)	0.761	7 (7.1%)	13 (13.3%)	0.157
Neoadjuvant therapy	3 (3.0%)	31 (3.7%)	1.000	3 (3.1%)	4 (4.1%)	1.000
Protective stoma	3 (3.0%)	36 (4.3%)	0.790	3 (3.1%)	1 (1.0%)	0.621
Laparoscopy	78 (78.8%)	622 (75.1%)	0.423	77 (78.6%)	78 (79.6%)	0.861
Tumor location			0.001*			0.227
Colon	29 (29.3%)	387 (46.7%)		29 (29.6%)	37 (37.8%)	
Rectum	70 (70.7%)	441 (53.3%)		69 (70.4%)	61 (62.2%)	
Tumor stage			0.078			0.936
I	29 (29.3%)	164 (19.8%)		28 (28.6%)	26 (26.6%)	
II	34 (34.3%)	345 (41.7%)		34 (34.7%)	36 (36.7%)	
III	36 (36.4%)	319 (38.5%)		36 (36.7%)	36 (36.7%)	

Variables are expressed as the mean ± SD, n (%), *P-value <0.05.

T2DM, type 2 diabetes mellitus; BMI, body mass index; PSM, propensity score matching; CHD, coronary heart disease.

### Univariate and Multivariate Analysis of OS and DFS

The medium follow-up time was 31 (1-113) months. Univariate analysis was conducted to find potential factors for prognosis and multivariate analysis was conducted to identify the independent predictors for prognosis.

**Figure 2:**
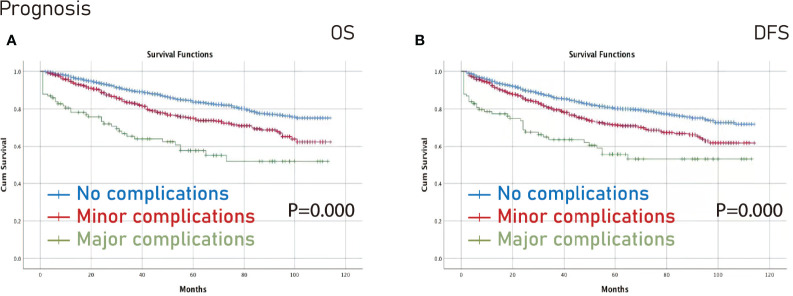
Prognosis among different complications groups. **(A)**, OS; **(B)**, DFS. OS, overall survival; DFS, disease-free survival.

The Kaplan-Meier curve was conducted to compare no complications/overall complications/major complications/minor complications. The no complications group had better OS (p=0.000) and DFS (p=0.000) than the minor complications group and the major complications group (**Figure 2**).

In terms of OS, age (p=0.000, HR=1.936, 95% CI=1.661-2.321), tumor stage (p=0.000, HR=2.088, 95% CI=1.844-2.364) and complications (p=0.000, HR=1.693, 95% CI=1.476-1.941) were independent predictors (**Table 3**).

**Table 3:** Univariate and multivariate analysis of overall survival.

Risk factors	Univariate analysis		Multivariate analysis	
	HR (95% CI)	P value	HR (95% CI)	P value
Age (>/≤64, years)	2.141 (1.817-2.522)	0.000*	1.963 (1.661-2.321)	0.000*
Sex (male/female)	0.897 (0.763-1.055)	0.188		
BMI (>/≤22.6)	0.793 (0.675-0.930)	0.004*	0.878 (0.747-1.032)	0.114
Hypertension (yes/no)	1.047 (0.874-1.255)	0.618		
T2DM (yes/no)	1.267 (1.005-1.598)	0.045*	1.055 (0.834-1.334)	0.657
Tumor site (colon/ rectum)	1.160 (0.990-1.359)	0.067		
Tumor stage (III/II/I)	2.073 (1.831-2.346)	0.000*	2.088 (1.844-2.364)	0.000*
Smoking (yes/no)	1.075 (0.914-1.264)	0.382		
Drinking (yes/no)	1.040 (0.876-1.234)	0.654		
CHD (yes/no)	1.299 (0.889-1.898)	0.177		
Complications (major/minor/none)	1.749 (1.530-1.999)	0.000*	1.693 (1.476-1.941)	0.000*

*P-value <0.05.

HR, Hazard ratio; CI, confidence interval; BMI, body mass index; T2DM, type 2 diabetes mellitus; CHD, coronary heart disease.

AS for DFS, age (p=0.000, HR=1.728, 95% CI=1.489-2.006), tumor stage (p=0.000, HR=2.047, 95% CI=1.830-2.291) and complications (p=0.000, HR=1.555, 95% CI=1.367-1.768) were independent predictors as well (**Table 4**).

**Table 4:** Univariate and multivariate analysis of disease-free survival.

Risk factors	Univariate analysis		Multivariate analysis	
	HR (95% CI)	P value	HR (95% CI)	P value
Age (>/≤64, years)	1.852 (1.598-2.146)	0.000*	1.728 (1.489-2.006)	0.000*
Sex (male/female)	0.902 (0.778-1.046)	0.172		
BMI (>/≤22.6)	0.854 (0.739-0.988)	0.034*	0.938 (0.810-1.085)	0.387
Hypertension (yes/no)	1.038 (0.880-1.224)	0.659		
T2DM (yes/no)	1.136 (0.914-1.412)	0.252		
Tumor site (colon/ rectum)	1.085 (0.939-1.254)	0.268		
Tumor stage (III/II/I)	2.039 (1.823-2.282)	0.000*	2.047 (1.830-2.291)	0.000*
Smoking (yes/no)	1.085 (0.936-1.257)	0.279		
Drinking (yes/no)	1.036 (0.886-1.211)	0.661		
CHD (yes/no)	1.228 (0.866-1.741)	0.250		
Overall complications (major/minor/none)	1.599 (1.410-1.814)	0.000*	1.555 (1.367-1.768)	0.000*

*P-value <0.05.

HR, Hazard ratio; CI, confidence interval; BMI, body mass index; T2DM, type 2 diabetes mellitus; CHD, coronary heart disease.

### Survival Analysis on Different Tumor Stages After PSM

**Figure 3:**
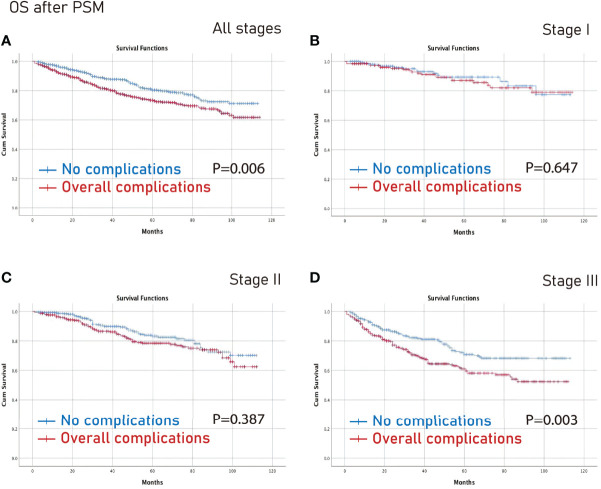
OS between the no complications group and the overall complications group after PSM. **(A)**, all stages; **(B)**, stage I; **(C)**, stage II; **(D)**, stage III. OS, overall survival; PSM, propensity score matching.

**Figure 4:**
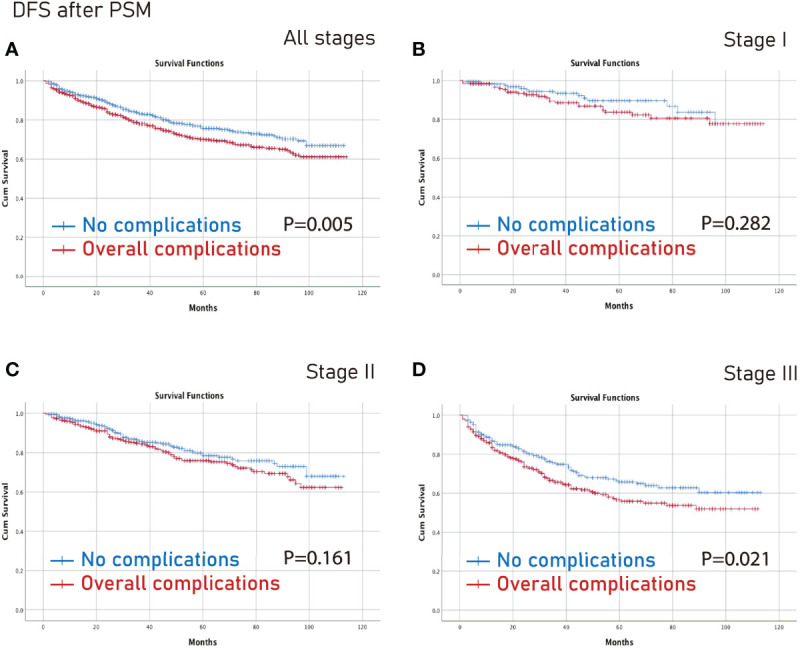
DFS between the no complications group and the overall complications group after PSM. **(A)**, all stages; **(B)**, stage I; **(C)**, stage II; **(D)**, stage III. DFS, disease-free survival; PSM, propensity score matching.

The no complications group had better OS on all stages (p=0.006) and stage III (p=0.003) CRC than the complications group after PSM (**Figure 3**). Furthermore, the no complications group had better DFS on all stages (p=0.005) and stage III (p=0.021) CRC than the complications group after PSM (**Figure 4**).

**Figure 5:**
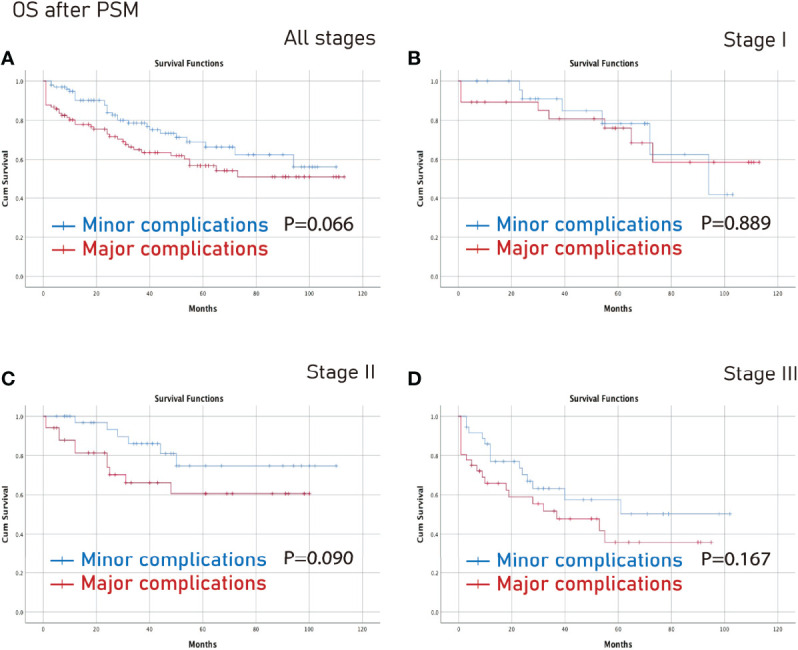
OS between the major complications group and the minor complications group after PSM. **(A)**, all stages; **(B)**, stage I; **(C)**, stage II; **(D)**, stage III. OS, overall survival; PSM, propensity score matching.

**Figure 6:**
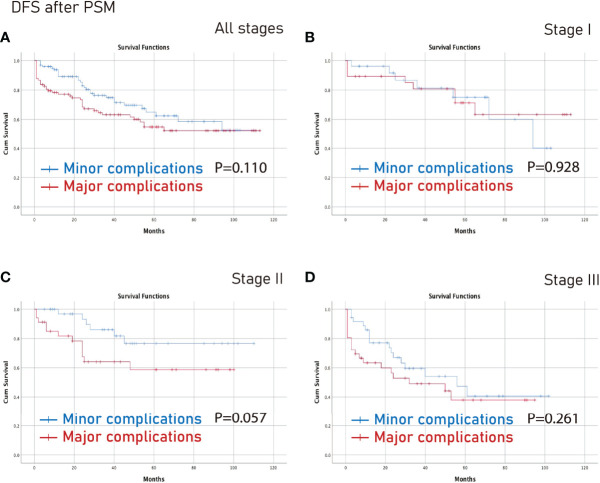
DFS between the major complications group and the minor complications group after PSM. **(A)**, all stages; **(B)**, stage I; **(C)**, stage II; **(D)**, stage III. DFS, disease-free survival; PSM, propensity score matching.

However, there was no significant difference between the minor complications group and the major complications group in terms of all stages, stage I, stage II or stage III (p>0.05) (**Figures 5**, **6**).

## Discussion

In this study, complications were independent predictors of OS and DFS. In terms of specific tumor stage, the no complications group had better OS on all stages and stage III CRC than the complications group after PSM. Furthermore, the no complications group had better DFS on all stages, and stage III CRC than the complications group after PSM. However, there was no significant difference between the minor complications group and the major complications group in different stages.

After CRC surgery, the incidence of complications was 18%-38% (6–8, 20–22). Complications could increase hospital stay and costs (9), furthermore, patients’ mental stress would increase, and some complications might be life-threatening. Therefore, surgeons should be cautious of complications and careful perioperative management was needed.

According to the Clavien-Dindo classification (19), complications could be divided into minor complications and major complications. Minor complications required observation, infusion and blood transfusion, and major complication required surgery, endoscopy or intervention. Various factors could result in complications including blood transfusion, T2DM and operation time (23, 24), moreover, complications could affect the prognosis (12–14).

Nowakowski M et al. (13) analyzed 265 CRC patients for three years and found that complications after laparoscopic CRC surgery had an impact on survival. Similarly, Slankamenac K et al. (14) reported that complications were associated with poorer long-term survival after surgery for CRC. However, Galata C et al. (15) found that neither overall complications nor major surgical complications were risk factors for decreased survival. The specific role of minor complications and major complications was not analyzed in previous studies. Furthermore, the PSM method was used to match the baseline information, which could minimize the selection bias. Besides, only one study reported the effect of complications on CRC using PSM (12), however, the included number of CRC patients was relatively small, and the specific role of minor complications and major complications was lacking in that PSM study.

To our knowledge, this is the first study to analyze the specific role of minor complications and major complications on different tumor stages of CRC using PSM.

There were many factors which could result in poor prognosis of CRC patients including tumor stage, age, BMI and T2DM (25–27). In the current study, we found that age, tumor stage and complications were independent factors of OS and DFS as well. The mechanism of complications which worsened the prognosis might be as follows: 1, complications might delay the follow-up chemotherapy of CRC patients, thus affecting OS and DFS (28, 29); 2, complications directly caused damage to the patients’ organs and inhibition of the adaptive immune response, resulting in malfunction, thus affecting OS and DFS (8, 30); and 3, postoperative peritoneal infection enhanced the invasive activity of residual tumor cells after CRC surgery (31).

In terms of specific CRC tumor stage, we found that OS was worse in stage III CRC patients, however, OS was not affected in stage I and II CRC. The mechanism was unclear, but stage III CRC patients might be more fragile to the delay of chemotherapy after CRC surgery.

This study had some limitations. First, this was a single-center retrospective study. Second, the follow-up time was relatively short in this study which might lead to inaccuracy of the outcomes. Therefore, multi-center, large-sample studies are needed in the following studies to clarify the exact relationship between complications and prognosis.

In conclusion, complications were associated with poor prognosis of CRC and surgeons should be cautious of the adverse events.

## Data Availability Statement

The datasets used and analyzed during the current study are available from the corresponding author on reasonable request.

## Ethics Statement

Ethical approval was obtained from the Institutional Ethics Committee of the First Affiliated Hospital of Chongqing Medical University (2021-537), and all patients signed informed consents. The patients/participants provided their written informed consent to participate in this study.

## Author Contributions

All authors contributed to data collection, BK and DP contributed to the data analysis; DP led the quality assessments; X-YL and BZ write the origin draft; Y-XC, CY, WT, and Z-QW revised the manuscript. All the authors have agreed on the manuscript which will be submitted, gave final approval of the version to be published, and agree to be accountable for all aspects of the work.

## Funding

This study was supported by Chongqing key diseases Research and Application Demonstration Program (Colorectal Cancer Prevention and Treatment Technology Research and Application Demonstration [No. 2019ZX003]).

## Conflict of Interest

The authors declare that the research was conducted in the absence of any commercial or financial relationships that could be construed as a potential conflict of interest.

## Publisher’s Note

All claims expressed in this article are solely those of the authors and do not necessarily represent those of their affiliated organizations, or those of the publisher, the editors and the reviewers. Any product that may be evaluated in this article, or claim that may be made by its manufacturer, is not guaranteed or endorsed by the publisher.

## References

[1] Sueda T Tei M Nishida K Yoshikawa Y Matsumura T Koga C. Impact of Prior Abdominal Surgery on Short-Term Outcomes Following Laparoscopic Colorectal Cancer Surgery: A Propensity Score-Matched Analysis. Surg Endosc (2021) 36(6):4429–41. doi: 10.1007/s00464-021-08794-3 34716479

[2] Bray F Ferlay J Soerjomataram I Siegel RL Torre LA Jemal A. Global Cancer Statistics 2018: GLOBOCAN Estimates of Incidence and Mortality Worldwide for 36 Cancers in 185 Countries. CA Cancer J Clin (2018) 68(6):394–424. doi: 10.3322/caac.21492 30207593

[3] Peng D Cheng YX Cheng Y. Improved Overall Survival of Colorectal Cancer Under Multidisciplinary Team: A Meta-Analysis. BioMed Res Int (2021) 2021:5541613. doi: 10.1155/2021/5541613 33997003 PMC8110396

[4] Wang S Wang X Zhou T Hu S Tian P Li Z. Effectiveness and Safety of Chinese Herbal Injections Combined With Fluoropyrimidine and Oxaliplatin-Based Chemotherapy for Advanced Colorectal Cancer: A Systematic Review and Meta-Analysis of 63 Randomized Controlled Trials. J Cancer (2021) 12(23):7237–54. doi: 10.7150/jca.60895 PMC8558662 34729124

[5] Cheng YX Tao W Zhang H Peng D Wei ZQ. Does Liver Cirrhosis Affect the Surgical Outcome of Primary Colorectal Cancer Surgery? A Meta-Analysis. World J Surg Oncol (2021) 19(1):167. doi: 10.1186/s12957-021-02267-6 34107967 PMC8191032

[6] Hendren S Birkmeyer JD Yin H Banerjee M Sonnenday C Morris AM. Surgical Complications are Associated With Omission of Chemotherapy for Stage III Colorectal Cancer. Dis Colon Rectum (2010) 53(12):1587–93. doi: 10.1007/DCR.0b013e3181f2f202 21178851

[7] Law WL Poon JT Fan JK Lo OS. Survival Following Laparoscopic Versus Open Resection for Colorectal Cancer. Int J Colorectal Dis (2012) 27(8):1077–85. doi: 10.1007/s00384-012-1424-8 PMC3401507 22318646

[8] Fujita Y Hida K Hoshino N Sakai Y Konishi T Kanazawa A. Impact of Postoperative Complications After Primary Tumor Resection on Survival in Patients With Incurable Stage IV Colorectal Cancer: A Multicenter Retrospective Cohort Study. Ann Gastroenterol Surg (2021) 5(3):354–62. doi: 10.1002/ags3.12433 PMC8164466 34095726

[9] Vonlanthen R Slankamenac K Breitenstein S Puhan MA Muller MK Hahnloser D. The Impact of Complications on Costs of Major Surgical Procedures: A Cost Analysis of 1200 Patients. Ann Surg (2011) 254(6):907–13. doi: 10.1097/SLA.0b013e31821d4a43 21562405

[10] Pang HY Zhao LY Wang H Chen XL Liu K Zhang WH. Impact of Type of Postoperative Complications on Long-Term Survival of Gastric Cancer Patients: Results From a High-Volume Institution in China. Front Oncol (2021) 11:587309. doi: 10.3389/fonc.2021.587309 34707984 PMC8542852

[11] Booka E Kikuchi H Hiramatsu Y Takeuchi H. The Impact of Infectious Complications After Esophagectomy for Esophageal Cancer on Cancer Prognosis and Treatment Strategy. J Clin Med (2021) 10(19):4614. doi: 10.3390/jcm10194614 34640631 PMC8509636

[12] Miyamoto Y Hiyoshi Y Tokunaga R Akiyama T Daitoku N Sakamoto Y. Postoperative Complications are Associated With Poor Survival Outcome After Curative Resection for Colorectal Cancer: A Propensity-Score Analysis. J Surg Oncol (2020) 122(2):344–9. doi: 10.1002/jso.25961 32346880

[13] Nowakowski M Pisarska M Rubinkiewicz M Torbicz G Gajewska N Mizera M. Postoperative Complications are Associated With Worse Survival After Laparoscopic Surgery for non-Metastatic Colorectal Cancer - Interim Analysis of 3-Year Overall Survival. Wideochir Inne Tech Maloinwazyjne (2018) 13(3):326–32. doi: 10.5114/wiitm.2018.76179 PMC6174179 30302145

[14] Slankamenac K Slankamenac M Schlegel A Nocito A Rickenbacher A Clavien PA. Impact of Postoperative Complications on Readmission and Long-Term Survival in Patients Following Surgery for Colorectal Cancer. Int J Colorectal Dis (2017) 32(6):805–11. doi: 10.1007/s00384-017-2811-y 28411352

[15] Osseis M Esposito F Lim C Doussot A Lahat E Fuentes L. Impact of Postoperative Complications on Long-Term Survival Following Surgery for T4 Colorectal Cancer. BMC Surg (2018) 18(1):87. doi: 10.1186/s12893-018-0419-y 30332994 PMC6192193

[16] Galata C Blank S Weiss C Ronellenfitsch U Reissfelder C Hardt J. Role of Postoperative Complications in Overall Survival After Radical Resection for Gastric Cancer: A Retrospective Single-Center Analysis of 1107 Patients. Cancers (Basel) (2019) 11(12):1890. doi: 10.3390/cancers11121890 PMC6966624 31783704

[17] Schofield JB Mounter NA Mallett R Haboubi NY. The Importance of Accurate Pathological Assessment of Lymph Node Involvement in Colorectal Cancer. Colorectal Dis (2006) 8(6):460–70. doi: 10.1111/j.1463-1318.2006.01044.x 16784464

[18] Weiser MR. AJCC 8th Edition: Colorectal Cancer. Ann Surg Oncol (2018) 25(6):1454–5. doi: 10.1245/s10434-018-6462-1 29616422

[19] Clavien PA Barkun J de Oliveira ML Vauthey JN Dindo D Schulick RD. The Clavien-Dindo Classification of Surgical Complications: Five-Year Experience. Ann Surg (2009) 250(2):187–96. doi: 10.1097/SLA.0b013e3181b13ca2 19638912

[20] Cohen ME Bilimoria KY Ko CY Hall BL. Development of an American College of Surgeons National Surgery Quality Improvement Program: Morbidity and Mortality Risk Calculator for Colorectal Surgery. J Am Coll Surg (2009) 208(6):1009–16. doi: 10.1016/j.jamcollsurg.2009.01.043 19476884

[21] Sjo OH Larsen S Lunde OC Nesbakken A. Short Term Outcome After Emergency and Elective Surgery for Colon Cancer. Colorectal Dis (2009) 11(7):733–9. doi: 10.1111/j.1463-1318.2008.01613.x 18624817

[22] Kwiatkowski AP Stępińska G Stanowski E Nesbakken A. Implementation of Laparoscopic Approach in Colorectal Surgery - A Single Center's Experience. Wideochir Inne Tech Maloinwazyjne (2018) 13(1):27–32. doi: 10.5114/wiitm.2018.72748 29643955 PMC5890849

[23] Wen J Pan T Yuan YC Huang QS Shen J. Nomogram to Predict Postoperative Infectious Complications After Surgery for Colorectal Cancer: A Retrospective Cohort Study in China. World J Surg Oncol (2021) 19(1):204. doi: 10.1186/s12957-021-02323-1 34238303 PMC8268384

[24] Jiang HH Dong XL Tang X Li AJ Chang Y Li HG. Nomogram for Predicting Risk of Intestinal Complications After Colorectal Cancer Surgery. Med Sci Monit (2019) 25:2104–11. doi: 10.12659/MSM.915692 PMC6439933 30897070

[25] Peng D Liu XY Cheng YX Tao W Cheng Y. Improvement of Diabetes Mellitus After Colorectal Cancer Surgery: A Retrospective Study of Predictive Factors For Type 2 Diabetes Mellitus Remission and Overall Survival. Front Oncol (2021) 11:694997. doi: 10.3389/fonc.2021.694997 34295822 PMC8290141

[26] Fu M Chen D Luo F Wang G Xu S Wang Y. Development and Validation of a Collagen Signature-Based Nomogram for Preoperatively Predicting Lymph Node Metastasis and Prognosis in Colorectal Cancer. Ann Transl Med (2021) 9(8):651. doi: 10.21037/atm-20-7565 33987349 PMC8106085

[27] Tang M Wang H Cao Y Zeng Z Shan X Wang L. Nomogram for Predicting Occurrence and Prognosis of Liver Metastasis in Colorectal Cancer: A Population-Based Study. Int J Colorectal Dis (2021) 36(2):271–82. doi: 10.1007/s00384-020-03722-8 32965529

[28] Krarup PM Nordholm-Carstensen A Jorgensen LN Harling H. Anastomotic Leak Increases Distant Recurrence and Long-Term Mortality After Curative Resection for Colonic Cancer: A Nationwide Cohort Study. Ann Surg (2014) 259(5):930–8. doi: 10.1097/SLA.0b013e3182a6f2fc 24045445

[29] McSorley ST Horgan PG McMillan DC. The Impact of the Type and Severity of Postoperative Complications on Long-Term Outcomes Following Surgery for Colorectal Cancer: A Systematic Review and Meta-Analysis. Crit Rev Oncol Hematol (2016) 97:168–77. doi: 10.1016/j.critrevonc.2015.08.013 26330375

[30] Artinyan A Orcutt ST Anaya DA Richardson P Chen GJ Berger DH. Infectious Postoperative Complications Decrease Long-Term Survival in Patients Undergoing Curative Surgery for Colorectal Cancer: A Study of 12,075 Patients. Ann Surg (2015) 261(3):497–505. doi: 10.1097/SLA.0000000000000854 25185465

[31] Salvans S Mayol X Alonso S Messeguer R Pascual M Mojal S. Postoperative Peritoneal Infection Enhances Migration and Invasion Capacities of Tumor Cells *In Vitro*: An Insight Into the Association Between Anastomotic Leak and Recurrence After Surgery for Colorectal Cancer. Ann Surg (2014) 260(5):939–43; discussion 943–4. doi: 10.1097/SLA.0000000000000958 25243554

